# News and Coming Events

**Published:** 1909-07-17

**Authors:** 


### NEWS AND COMING EVENTS.

SINCE June 12, 1909, the Royal Sanitary Institute and Parkes Museum, has been removed to No. 90 Buckingham Palace Road, London, S.W.

THE total amount received up to July 12 at the Mansion House on behalf of the Metropolitan Hospital-Sunday Fund for this year was approximately £40,000.

THE Children's Sanatorium at Holt, Norfolk, for the treatment of phthisis, has just received a gift of £300 "In memoriam M. T." The amount will be placed to the fund for the permanent building which it is proposed to proceed with when the necessary amount is reached. It is understood that some six thousand pounds is required.

THE Leyton Urban District Ratepayers' Association has arranged to affix a memorial tablet to the birthplace of Sir Morell Mackenzie, 742 High Road, Leytonstone. The premises are at the corner of Browning Road, near the tram terminus and within five minutes of Leytonstone G.E. Railway station. The tablet will be unveiled on Monday, July 19, at 7.10 P.M.

THE third Norman Kerr Lecture of the Society for the Study of Inebriety will be delivered by Professor Taav. Laitinen, M.D., Professor of Hygiene and Director of the Hygienic Institute in the University of Helsingfors, Finland, on Tuesday next, July 20, at 8 P.M., in the lecture theatre of the Victoria and Albert Museum, South Kensington. The subject of the lecture will be "The Influence of Alcohol on Immunity."

THE Committee of the Royal National Orthopædic Hospital, 234 Great Portland Street, London, W. (with which is incorporated the City Orthopædic Hospital), announces that His Majesty the King has consented formally to open, at 12 o'clock on Friday, July 23, the new hospital which has just been completed in Great Portland Street. His Majesty thus shows his appreciation of the important amalgamation of the three Orthopædic Hospitals which formally existed in London. This amalgamation was carried through, after much negotiation, as the result of the policy inaugurated by the King's Hospital Fund Committee. Over £20,000 is still needed to complete the £75,000 which the new hospital has cost.

THE body of the late Dr. Cowas Lalcaca, M.D.Brux., L.R.C.P.Lond., L.M.S.Bombay, who was shot by an Indian assassin while endeavouring to protect from the same fate Sir Curzon Wyllie at the reception at the Imperial Institute on Thursday, July 1, was buried in Brookwood Cemetery. There was a large attendance of prominent Indian residents in London, and Viscount Morley, Secretary of State for India, was represented. The coffin bore the inscription "Dr. Cowas Lalcaca, of Shanghai and Bombay, died July 1, 1909, aged 48 years," and was accompanied by many beautiful wreaths. Lady Wyllie's tribute bore the inscription: "With deepest regrets. These flowers are sent by the wife of Sir Curzon Wyllie in ever-grateful remembrance of the brave and noble man who lost his life on the night of July 1 in trying to save her beloved husband and others. With deepest sympathy." At Brookwood the coffin was carried to a mausoleum in the Parsee burying ground. The procession to the grave was headed by Parsee ladies and a large number of Indian and English gentlemen. At the mausoleum a short service was conducted by Mr. R. Desai, and the body was buried according to the rites of the Zoroastrian religion.

THE Chelsea Hospital for Women has lately received from Lady Tate the sum of £1,000 for the purpose of endowing a bed.

THE opening ceremony of the Convalescent Home of the Royal Edinburgh Hospital for Sick Children at Muirfield House, Gullane, takes place to-day (Saturday, July 17) at 3 o'clock P.M. A train leaves Edinburgh for Gullane at 1.35 P.M., returning from Gullane at 4.13 P.M. and 5.30 P.M.

PROFESSOR E. H. STARLING, M.D., F.R.S., president of Section I. (Physiology) of the British Association, has selected as the title of his presidential address "The Physiological Factors of Success." It will deal with the mechanisms which have been adapted by the animal kingdom in their struggle for existence and have led to rise of type—*i.e.*, increased range of adaptation.

AT a meeting of the West India Committee last week it was decided, on the suggestion of the Chairman, Mr. Owen Philipps, M.P., to send the following telegram to Mr. Chamberlain: "Mindful of all you have done for the West Indies and the Colonies and the interest you take in the School for Tropical Medicine, the members of the West India Committee desire to express cordial greetings to you on the occasion of your birthday."

THE Duke of Devonshire distributed the prizes last week to the successful students at Guy's Hospital Medical School, when Mr. Cosmo Bonsor, the Treasurer of the hospital, presided. The report of the Dean of the Medical School drew especial attention to the undoubted fact that at the present time the demand for qualified men far exceeds the supply, and that therefore the medical profession now holds out improved prospects to boys on leaving school.

THE National Food Reform Association informs us that it has now opened offices at 178 St. Stephen's House, Westminster, S.W., overlooking the Houses of Parliament. The Association has lately published a penny booklet entitled "Economical Dishes for Workers" suitable for district visitors and social workers. A specimen copy, with full particulars regarding the Association, may be obtained by sending a stamped addressed envelope to the Secretary at the above new address.

AT the Festival Dinner held in aid of the funds of the Charing Cross Hospital at the Whitehall Rooms on July 13, under the presidency of the Lord Mayor of London, it was announced that £2,613 has been received. In responding to the toast of "Success to the Hospital," proposed by the Lord Mayor, Viscount Ridley, the Chairman of the hospital, appealed to the generous public for help in reducing the outstanding mortgage of £85,000, interest and sinking fund charges upon which are a severe handicap to the committee. He explained also that since the last festival dinner in 1906 £15,000 of debt other than mortgage has been wiped out, and the overdraft of £5,000 at the bank converted into a balance of £1,100. The annual expenses have been reduced from £26,000 to £23,500 without any diminution of efficiency. These improvements in the financial position of the hospital have been partly owing to some exceptional legacies, and Lord Ridley appealed especially for extra subscribers, so as to render the committee less dependent on such windfalls, which may not improbably be diminished in the near future as the result of increased death duties.

It is officially announced that his Majesty the King has consented to lay the foundation-stone of the new King's College Hospital buildings at Denmark Hill at twelve o'clock noon on Tuesday next, July 20.

On Saturday last, July 10, which was Degree Day at the University of Liverpool, Lord Derby, the Chancellor, conferred the degree of M.Ch. on Mr. Robert Jones and Mr. William Thelwall Thomas, two eminent Liverpool surgeons.

Mr. John William Day, woollen manufacturer, of Harrogate, who died last April, out of an estate valued at 132,564*l.*, bequeathed 1,000*l.* to the Huddersfield Infirmary for the purpose of founding a John William Day bed, and 100*l.* to the Harrogate Infirmary.

At the first meeting of the council of the University of Bristol, on July 9, Dr. Lloyd Morgan tendered his resignation of the office of Vice-Chancellor, and Sir Isambard Owen, Principal of Armstrong College, Newcastle-on-Tyne, was elected Vice-Chancellor as from September 9 next.

The late Dr. Percy Boulton, M.D., consulting physician to the Samaritan Hospital for Women and Children and to the British Home for Incurables, left estate valued at 20,520*l.* gross. He bequeathed 100*l.* to the Samaritan Free Hospital for Women, Marylebone Road.

The Prince of Wales as Grand Prior of the Order of St. John of Jerusalem, at Marlborough House, on July 9, presented the awards of the Order granted during the past year for saving life, and the Service Medals awarded for conspicuous services to the Order. His Royal Highness afterwards presided at the annual meeting at Marlborough House of the General Committee of the Imperial Cancer Research Fund.

On Monday evening last, July 12, in the board room of the London Temperance Hospital, under the presidency of Alderman Sir T. Vezey Strong, Chairman of the Board of Management, a portrait of Dr. Dawson Burns was formally presented to the hospital. Dr. Burns was Hon. Secretary of the Provisional Committee in 1871-73, and has been the Hon. Secretary of the institution from 1873 to the present time. The portrait, which is considered an excellent and artistic likeness, was executed by Mr. Cecil Lawrence Burns, a younger son of Dr. Burns, and Principal of the Bombay School of Art, and it was offered by the artist to the board of management, on whose behalf it was gratefully accepted by Sir T. Vezey Strong. The company consisted of managers and various prominent supporters of the institution.

The eighth International Congress of Tuberculosis was opened at Stockholm on July 8 in the Chamber of the Upper House of the Riksdag. M. Bourgeois, of Paris, presided, and the company included Prince Carl, Princess Ingeborg, and Prince Eugène, and there were also present delegates and representative scientists of all nations. Dr. Hope, representing the City and University of Liverpool, was at first the only one of the British delegates, while Dr. Theodore Williams was detained in Berlin by an accident. The deliberations of the Congress were especially concerned with preventive measures and systems of fresh-air treatment, and the injection of tuberculin. Surgeon Wise, medical director of the American Navy, strongly advocated legislation for the compulsory registration and segregation of tuberculous persons. At the termination of the Conference on July 10 the President expressed general gratitude to the King and Queen of Sweden and the city of Stockholm for their encouragement, co-operation, and hospitality, and Baron Tamm, President of the Swedish Anti-Tuberculosis Society, responded. The next conference will meet in Brussels.

The Duchess of Albany presided at a meeting of the Executive Committee of the Jubilee Fund of the National Hospital for the Paralysed and Epileptic, Queen Square, on July 8, for the purpose of making arrangements for the visit of the Princess of Wales on Saturday, October 9, on which occasion Her Royal Highness will receive purses of 10*l.* and upwards. It is understood that donors and collectors of such purses will receive invitations to be present when the King opens the Jubilee extension of the hospital on November 4. The Secretary of the National Hospital will be glad to hear from any lady or gentleman willing to provide a purse.

### MEDICAL OFFICERS AND THE POOR LAW COMMISSION.

The annual meeting of the Poor Law Medical Officers' Association of England and Wales was held at the Guildhall on July 6, under the presidency of Surgeon-General Evatt. The Lord Mayor and Sheriff Sir J. Baddeley extended an official welcome to the members, and in his reply the chairman deplored that at the present time there is no Minister of Health for England, whose duty it would be to speak for the medical profession as a whole and advise the Government on health questions. Dr. Major Greenwood, district medical officer of Shoreditch, read a paper on "District Medical Officers and the Report of the Poor Law Commission," and "Medical Relief and Public Assistance" formed the subject of a communication by Mr. C. S. Loch, secretary of the Charity Organisation Society. Mrs. Sidney Webb read a paper on "The Place of the District Medical Officer in a Unified County Medical Service." Dr. Smith-Whittaker, the medical secretary of the British Medical Association, criticised the proposals of the Majority Report of the Commission, and in the afternoon a paper on "The Genesis of the Poor Law Infirmary" was read by Dr. F. S. Toogood, medical superintendent of the Lewisham Infirmary. Dr. G. F. McCleary, medical officer of health for Hampstead, read a paper on "The Public Health Aspect of the Royal Commission," and said that the real object of the Majority seemed to be the establishment of a State-aided insurance against sickness. Free medical relief would be afforded without adequate safeguards for the interests of either the medical profession or the ratepayers. In the discussion on the two papers Surgeon-General Evatt declared that the question of public health ought not to be considered solely from the point of view of the rates. On the motion of Dr. Major Greenwood, seconded by Dr. Napper, it was resolved that in any system of Poor Law medical reform there must be a distinct service for the sick State poor, so that they may be treated with due regard to economy, and to prevent pauperisation by granting free medical attendance to a large number of the community. It was also resolved that in sparsely inhabited rural districts, where access to the relieving officer is difficult, all persons requiring State aid shall apply to a special officer, who shall decide as to the right of the applicant to help.

Surgeon-General Evatt was re-elected president, Dr. D. B. Balding as chairman of Council, Dr. Napper as hon. treasurer, Dr. Lloyd Brown as hon. auditor, and Dr. Major Greenwood as hon. secretary for the ensuing year.

The annual dinner, held at the Waldorf Hotel, was also presided over by Surgeon-General Evatt, who was supported by Sir Walter Foster, M.P., Sir Dyce Duckworth, Alderman Sir Thomas Crosby, Sir Shirley and Lady Murphy, Prebendary Russell Wakefield, Mr. T. R. Ferens, M.P., Dr. Downes, the Mayor of Finsbury (Dr. W. A. Dingle), Mr. C. S. Loch, Mr. and Mrs. Sidney Webb, and Dr. M. Greenwood (hon. secretary).

